# Supplementary material for: Comparative genomics provides new insights into the diversity, physiology, and sexuality of the only industrially exploited tremellomycete: Phaffia rhodozyma
Source: BMC Genomics. 2016 Nov 9;17:901. doi: 10.1186/s12864-016-3244-7 (PMC5103461; doi:10.1186/s12864-016-3244-7)
Supplement: Additional file 6: — List of orphan genes with links to PFAM (related to Additional file 1: Table S1). (ZIP 1428 kb) [file 12864_2016_3244_MOESM6_ESM.zip › PFAM_UNIQUE/G05584_P.html]

#                                                                            --- full sequence --- -------------- this domain -------------   hmm coord   ali coord   env coord
  
# target name        accession   tlen query name           accession   qlen   E-value  score  bias   #  of  c-Evalue  i-Evalue  score  bias  from    to  from    to  from    to  acc description of target
  
#------------------- ---------- ----- -------------------- ---------- ----- --------- ------ ----- --- --- --------- --------- ------ ----- ----- ----- ----- ----- ----- ----- ---- ---------------------
  
G05584\_P             -            143 Ashwin               PF15323.1    215      0.75    8.4   4.4   1   1    0.0011      0.93    8.1   4.4   123   208    41   134    10   139 0.53 - Developmental protein  
G05584\_P             -            143 DUF3446              PF11928.3     84       2.9    6.9  11.5   1   2    0.0019      0.68    8.9   8.5    35    53    47    65     8    84 0.61 - Domain of unknown function (DUF3446)  
G05584\_P             -            143 DUF3446              PF11928.3     84       2.9    6.9  11.5   2   2       7.8   2.7e+03   -2.7   0.0    45    49   107   111    94   124 0.54 - Domain of unknown function (DUF3446)  
G05584\_P             -            143 GATA-N               PF05349.7    179     0.068   12.0   1.6   1   1   2.7e-05     0.081   11.8   1.6    68   102    48    82    12   120 0.72 - GATA-type transcription activator, N-terminal  
G05584\_P             -            143 MYT1                 PF08474.6    257       8.1    4.5   8.0   1   2    0.0027       5.4    5.1   5.9    65    98    38    69     9    76 0.64 - Myelin transcription factor 1  
G05584\_P             -            143 MYT1                 PF08474.6    257       8.1    4.5   8.0   2   2      0.19   3.8e+02   -1.0   0.0   177   202    94   119    79   134 0.68 - Myelin transcription factor 1  
G05584\_P             -            143 Period\_C             PF12114.3    195       8.2    4.6   8.3   1   1     0.019        13    4.0   8.3    23    58    36    67     8    76 0.55 - Period protein 2/3C-terminal region  
G05584\_P             -            143 SOG2                 PF10428.4    445         4    4.6   6.5   1   1      0.02       4.3    4.5   6.5   179   216    34   102     3   140 0.38 - RAM signalling pathway protein  
G05584\_P             -            143 SSP160               PF06933.6    756       7.7    3.0   8.8   1   1     0.044       9.4    2.7   8.8   102   128    42    68    29    85 0.61 - Special lobe-specific silk protein SSP160
